# *MTH1* and *RGT1* demonstrate combined haploinsufficiency in regulation of the hexose transporter genes in *Saccharomyces cerevisiae*

**DOI:** 10.1186/1471-2156-13-107

**Published:** 2012-12-12

**Authors:** Kevin L Dietzel, Vidhya Ramakrishnan, Erin E Murphy, Linda F Bisson

**Affiliations:** 1 Department of Viticulture and Enology, University of California, Davis, Davis, CA, 95616, USA

**Keywords:** Haploinsufficiency, Glucose signaling, *Snf3* suppressor, RGT1, MTH1, SNF3, Saccharomyces

## Abstract

**Background:**

The *SNF3* gene in the yeast *Saccharomyces cerevisiae* encodes a low glucose sensor that regulates expression of an important subset of the hexose transporter (HXT) superfamily. Null mutations of *snf3* result in a defect in growth on low glucose concentrations due to the inability to relieve repression of a subset of the *HXT* genes. The *snf3* null mutation phenotype is suppressed by the loss of either one of the downstream co-repressor proteins Rgt1p or Mth1p. The relief of repression allows expression of *HXT* transporter proteins, the resumption of glucose uptake and therefore of growth in the absence of a functional Snf3 sensor.

**Results:**

Strains heterozygous for both the *RGT1* and *MTH1* genes (*RGT1/rgt1Δ MTH1/mth1Δ snf3Δ/snf3Δ*) but homozygous for the *snf3*∆ were found to grow on low glucose. Since null alleles in the heterozygous state lead to suppression, *MTH1* and *RGT1* display the phenomenon of combined haploinsufficiency. This observed haploinsufficiency is consistent with the finding of repressor titration as a mechanism of suppression of *snf3*. Mutants of the *STD1* homolog of *MTH1* did not display haploinsufficiency singly or in combination with mutations in *RGT1*. *HXT* gene reporter fusion assays indicated that the presence of heterozygosity at the *MTH1* and *RGT1* alleles leads to increased expression of the *HXT2* gene. Deletion of the *HXT2* gene in a heterozygous diploid, *RGT1/rgt1Δ MTH1/mth1Δ snf3Δ/snf3Δ hxt2Δ/hxt2Δ,* prevented the suppression of *snf3Δ*.

**Conclusions:**

These findings support the model of relief of repression as the mechanism of restoration of growth on low glucose concentrations in the absence of functional Snf3p. Further, the observation that *HXT2* is the gene responsible for restoration of growth under these conditions suggests that the numbers of repressor binding domains found in the regulatory regions of members of the *HXT* family may have biological relevance and enable differential regulation.

## Background

Glucose is the preferred carbon source for many organisms including the budding yeast *Saccharomyces cerevisiae*. Glucose transport occurs via facilitated diffusion mediated by a group of homologous transmembrane hexose transporters, encoded by the *HXT* genes [1]. Transport is the rate limiting step in glucose utilization by *S. cerevisiae* and a complex regulatory network has evolved to maintain optimal transporter activity in response to external nutrient availability [1,2]. Many signaling pathways are known to converge at the promoters of the *HXT* genes to either positively or negatively regulate specific transporters in response to their respective inputs. These pathways include: glucose induction, glucose repression, High Osmolarity Glycerol (HOG), Target of Rapamycin (TOR) GCR1/GCR2 pathway and the RAS/PKA pathway [3-8].

Although interpathway crosstalk is likely crucial to optimized *HXT* regulation, it is clear that the relief of *HXT* repression via the *SNF3/RGT2* glucose induction pathway is the major on/off switch essential to transporter induction. Mutations in several components of this pathway show extreme growth defects on glucose that result from the inability to induce high level expression of the major *HXT* genes [9-11]. Snf3p and Rgt2p, which display homology to the *HXT* transporters, are thought to act as sensors of low and high glucose respectively [12-14]. In contrast to the *HXT's*, both Snf3p and Rgt2p appear to have lost the capacity to transport glucose and both proteins also contain an extra C terminal cytoplasmic domain that is important for the transmission of the signal [14]. Null alleles of *snf3* result in the loss of fermentative growth on low levels of glucose (0.05%) while null alleles of both sensors result in the loss of fermentative growth on 2% glucose [11,15,16].

Mth1p and Std1p are the only known link between the cytoplasmic membrane bound sensors and the DNA binding repressor Rgt1p in the nucleus where they act to facilitate the repressor function of Rgt1p [11,17,18]. Rgt1p appears to regulate a limited set of genes including most of the *HXT* genes that are critical for growth on glucose [12,13,19]. It is not known how glucose specifically activates the sensors, but activation requires the cytoplasmic domains which are thought to facilitate the phosphorylation of the homologous corepressor proteins Mth1p and Std1p by the Yeast Casein Kinase (*YCK1*, *YCK2*) [11,14,16,20]. Phosphorylation targets the corepressors for ubiquitination which results in their degradation [17]. Degradation of Mth1p and Std1p is thought to inactivate the repressor function of Rgt1p and allow for its phosphorylation by the Protein kinase A homologues [7,8,17,19]. As a result, the promoters of the *HXT* genes are then open for transcription.

Although a set of key proteins have been identified and a working model of the glucose induction pathway exists, many questions remain about how these components interact and function to regulate *HXT* gene expression in response to external glucose levels. During complementation analyses of repressor mutants it was discovered that diploid strains homozygous for the *snf3* null mutation and heterozygous at both the *MTH1* and *RGT1* loci (*snf3Δ/snf3Δ MTH1/mth1 RGT1/rgt1*) were able to grow on low glucose. Haploinsufficiency arises when a reduction in copy number results in an observable phenotype [21]. The appearance of a phenotype in the presence of recessive alleles of different genes has been termed combined haploinsufficiency [22]. Combined haploinsufficiency arises when reduction in dosage of one gene displays a mutant phenotype only in the presence of an accompanying reduction in dosage of a second gene. This genetic phenomenon is rare and typically implies that the proteins involved form a complex and that loss of a critical concentration of the complex is responsible for the observed phenotype. This observation indicates that the levels of both of these proteins are in a critical balance with the number of promoters that they regulate. The impacts of this finding on the current model of the glucose induction pathway are discussed.

## Results and discussion

### MTH1 *and* RGT1 *display combined haploinsufficiency*

The *MTH1* gene encodes a co-repressor protein that is able to bind to the repressor *RGT1* stabilizing binding of the complex to the regulatory regions of the *HXT* genes. In the presence of a glucose signal mediated by either Snf3p or Rgt2p Mth1p is phosphorylated and targeted for ubiquitination and Rgt1-mediated repression is inactivated. Deletion of either *MTH1* or *RGT1* restores expression of the *HXT* genes as the repressor complex cannot be established. Loss of either of these genes is recessive to the wild type, indicating that in a diploid situation only a single functioning repressor gene is necessary.

In the course of analysis of missense mutations in *rgt1* and *mth1* we observed suppression of the *snf3Δ* growth defect on low glucose in strains that were heterozygous at these loci (*snf3Δ/snf3Δ RGT1/rgt1 MTH1/mth1*). This finding suggested one of two possibilities for the mechanism of suppression: non-allelic non complementation or combined haploinsufficiency. In non-allelic non complementation the appearance of a phenotype is due to the creation of an aberrant regulatory complex that in this case would presumably be leading to activation of expression. On the other hand, combined haploinsufficiency occurs when the decrease in gene dosage of two genes with interacting gene products results in a dynamic reduction in the level of the complex itself and expression of the mutant phenotype. Suppression in this case would be mediated by relief of repression due to the decrease in repressor complex availability. Combined haploinsufficiency is a rare genetic phenomenon that implies physical interaction of the gene products [22]. These two phenomena can be distinguished by investigation of the phenotype of null mutations. If suppression still exists in the presence of heterozygous null mutations then loss of repressor complex rather than formation of an aberrant regulatory complex is the mechanism by which growth is restored to *snf3Δ* strains.

To test this possibility, complete null alleles were created of both *mth1* and *rgt1* in the *snf3Δ* background (UCD2875 and UCD2876, respectively). Both mutations independently suppressed the *snf3*Δ growth defect in haploid strains as expected and both acted as fully recessive mutations in homozygous diploids also as expected. However heterozygous diploids at both of these loci (*RGT1/rgt1Δ snf3Δ/snf3Δ and MTH1/mthΔ snf3Δ/snf3Δ*) (UCD2881) failed to complement and growth on low glucose occurred indicating that combined haploinsufficiency is the genetic phenomenon underlying apparent suppression of the *snf3Δ* phenotype.

**Figure 1:**
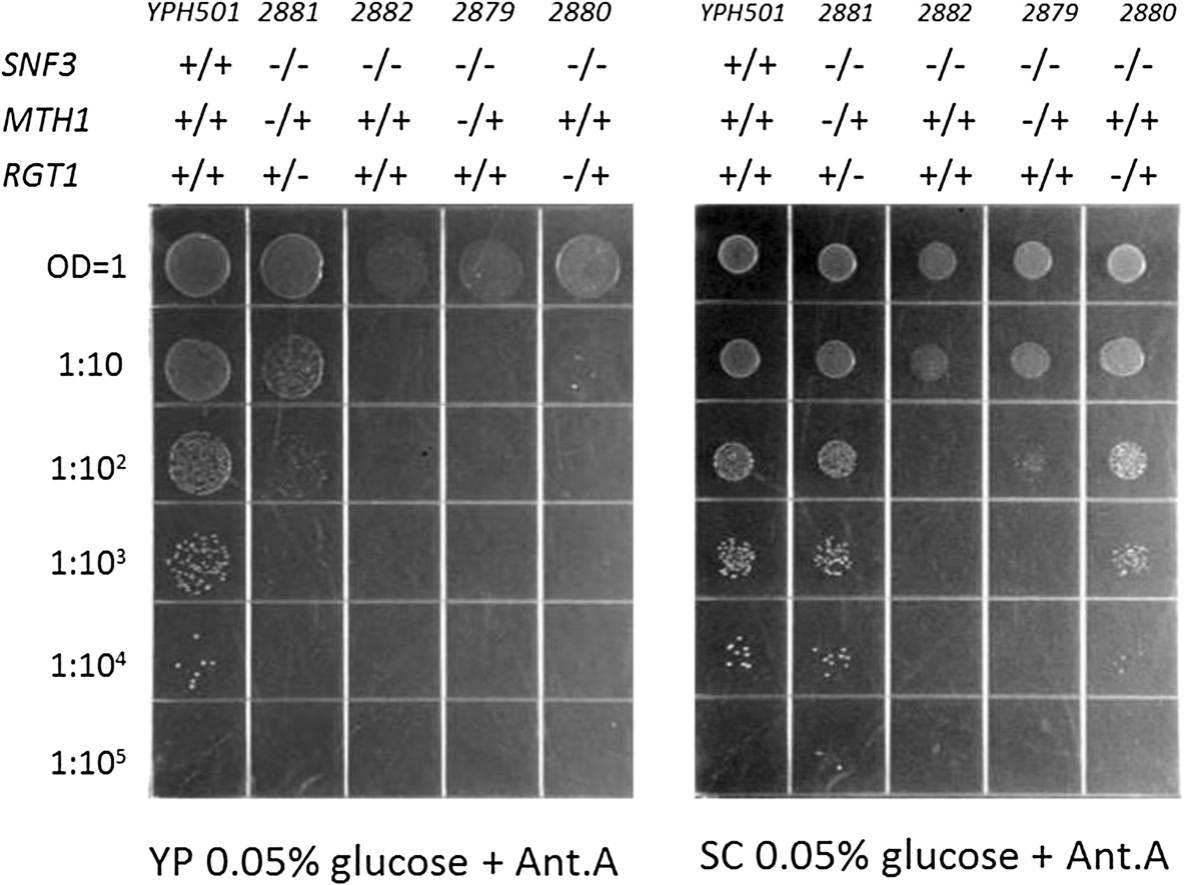
**Spot plate assay of strains on low glucose reveals combined haploinsuficiency on YP 0.05% glucose + Antimycin A (left) and combined and single haploinsufficiency on SC 0.05% glucose + Antimycin A (right).** The strains used are wild type YPH501 (column 1), *snf3/snf3 mth1/MTH1 rgt1/RGT1* (UCD2881) (column 2), *snf3/snf3* (UCD2882) (column 3), *snf3/snf3 mth1/MTH1* (UCD2879) (column 4), and *snf3/snf3 rgt1/RGT1* (UCD2880) (column 5).

Spot plate assays were used to further refine the growth patterns of heterozygous diploid strains (Figure 1). The double heterozygote *snf3/snf3 mth1/MTH1 rgt1/RGT1* (UCD2881) exhibits suppression of the *snf3* null phenotype (column 2, Figure 1). Growth of the double heterozygote on YP 0.05% glucose does not appear to be as robust as wild type (YPH501) (column 1, Figure 1) but is clearly better than *snf3* null (UCD2882) (column3) or either of the single heterozygotes *snf3/snf3 mth1/MTH1* (UCD2879) (column 4) or *snf3/snf3 rgt1/RGT1* (UCD2880) (column 5). The single heterozygotes appear to be completely recessive on YP media. However, on SC media (Figure 1), growth of the double heterozygote appears to be equivalent to wild type and also reveals that both of the single heterozygotes (columns 4 and 5) show some degree of single haploinsufficiency compared to the *snf3/snf3* control (column 3). This single haploinsufficiency is much more pronounced in the *snf3/snf3 rgt1/RGT1* strain than in the *snf3/snf3 mth1/MTH1* strain.

### *Loss of the* MTH1 *homolog* STD1 *does not suppress loss of* SNF3

*STD1* has been reported to be somewhat functionally redundant to *MTH1* and to be transcriptionally regulated by the *RGT1* repressor complex similar to the *HXT* genes [19,23]. This “feed forward” regulation has been proposed to act to quickly restore the repressor complex as glucose levels drop [7]. Therefore the role of *STD1* in suppression of the *snf3* growth defect was evaluated.

**Figure 2:**
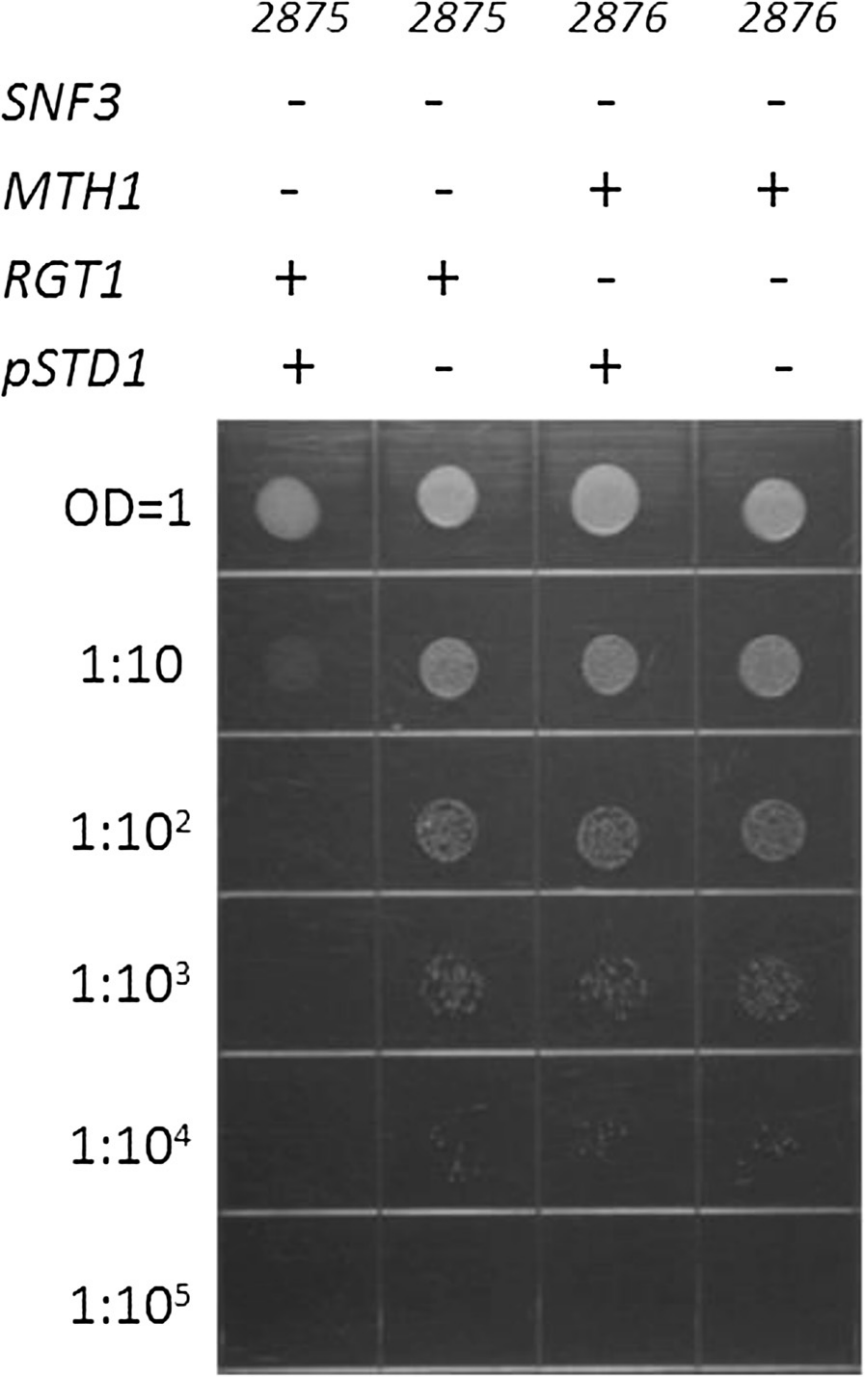
**The overexpression of *STD1 *from a strong constitutive promoter prevents the suppression of the *snf3 *null growth defect on low glucose by *mth1 *null and results in no growth on low glucose of *snf3 mth1 *(UCD2875) (column 1).** The vector only control in the same strain can grow (column 2). Overexpression of *STD1* does not prevent the suppression of the *snf3* null in *snf3 rgt1* (UCD2876) (column 3) indicating the requirement of *RGT1* for *STD1*function. The vector only control in *snf3 rgt1* (UCD2876) is shown in (column 4).

The complete null allele of *std1* was created in the *snf3* null background (UCD2877 and UCD2978). Loss of *std1* did not suppress the *snf3* null phenotype and *snf3/snf3 std1/std1* grew exactly as *snf3/snf3* in a spot plate assay (data not shown). To ensure that the *STD1* allele is functional in our strain background, the *STD1* open reading frame was cloned into a vector under the control of a strong constitutive promoter (TEF) and transformed into a haploid *snf3 mth1* strain and a haploid *snf3 rgt1*strain. Overexpression of active Std1p leads to a restoration of repression to the *HXT* genes and suppression of loss of *mth1* since Std1p is able to bind to Rgt1p and thence protect repressor complex binding to the promoters in the absence of Mth1p. Strong overexpression of *STD1* restored the active repressor complex to a *snf3 mth1*strain preventing growth of the strain on low glucose while the same strain with the vector only is able to grow (Figure 2). However, a *snf3 rgt1* strain overexpressing *STD1* can grow confirming that *STD1*-mediated regulation requires the Rgt1 co-repressor (Figure 2).

Thus *STD1* did not show combined haploinsufficiency in the presence of heterozygosity at the *RGT1* locus. Due to the significant amount of sequence identity (61%) and somewhat overlapping yeast two hybrid interactions with the tail domains of the glucose sensors and also with Rgt1p, the corepressor proteins Mth1p and Std1p have been described in a manner that implies that they are functionally redundant or at least have overlapping functions [23,24]. In contrast, other studies have concluded that Mth1p is likely to be more specific to the regulation of *HXT* genes than Std1p [11,16,17] but agree that some data suggests enhanced derepression when both genes are deleted. Our analysis suggests that Mth1p alone is primarily responsible for maintaining repression downstream of Snf3p when glucose levels are limiting*.* In addition, our finding that deleting *std1* along with *mth1* does not augment adaptation to low glucose and that the deletion of *std1* shows no detectable effect on the adaptation of *snf3* null strains to low glucose lead us to conclude that Std1 has little effect in regulating Rgt1p function under the low glucose conditions used in this study. However, our observation that the overexpression of *STD1* from a strong promoter prevents the suppression of the *snf3* growth defect by an *mth1* null mutant and that this requires a functional Rgt1p corroborates the conclusion that Std1 can act to prevent growth on low glucose, but implies that it may not normally do so. The discovery that the [GAR+] prion allows growth on alternate substrates in the presence of glucose due to a specific interaction of the Pma1p and Std1p proteins fostering the creation of a stable Std1p-Rgt1p complex [25] strongly supports the idea that Std1p regulates *HXT* gene expression under specific physiological conditions.

### Low glucose growth curves confirm combined and single haploinsufficiency

While the loss of *snf3* function results in the inability to grow on low glucose solid media, it results in a dramatic increase in the time it takes to adapt to low glucose liquid media and a diminished capacity to achieve the same final OD of wild type strains [14]. The spot plate assays suggested that there may be differences in adaptation times due to the single and combined haploinsufficiency observations.

**Figure 3:**
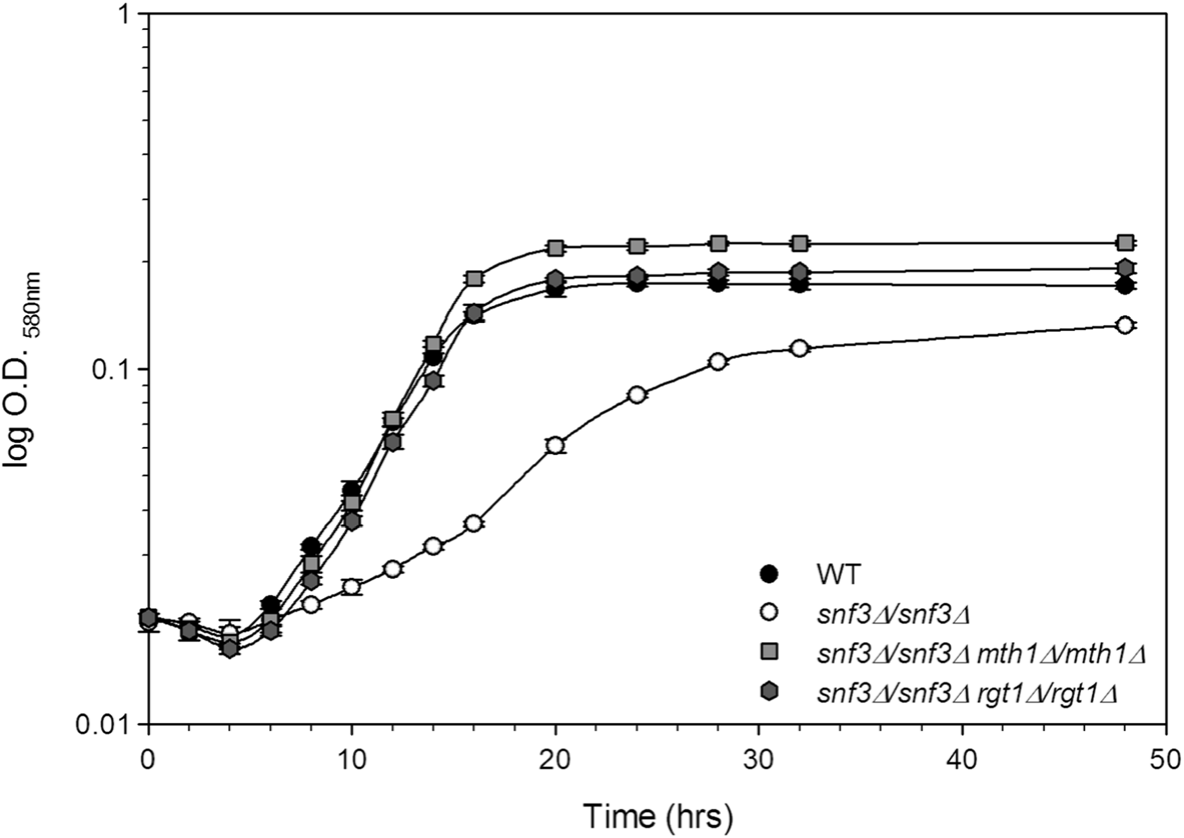
**Low glucose growth curves show that homozygous recessive mutations in *mth1 *and *rgt1 *grow equivalent to wild type.** Panel A: Wild Type (WT) (YPH501), *snf3/snf3* (UCD2882), *snf3/snf3 mth1/MTH1 rgt1/RGT1* (UCD2881), *snf3/snf3 mth1/MTH1* (UCD2879), and *snf3/snf3 rgt1/RGT1* (UCD2880). Panel B: Wild Type (WT) is YPH501, *snf3/snf3* (UCD2882), *snf3/snf3 mth1/mth1* (UCD2883), snf3*/snf3 mth1/mth1 rgt1/rgt* (UCD2886), and *snf3/snf3 mth1/mth1 std1/std1* (UCD2888).

The growth curves of homozygous recessive diploids of *rgt1* and *mth1* in the *snf3* null strains were examined to determine if these mutations can completely suppress the adaptation defect. The adaptation time of the *snf3/snf3 mth1/mth1* (UCD2883)*, snf3/snf3 rgt1/rgt1* (UCD2884) strains and wild type (YPH501) are equivalent (Figure 3A). This not only suggests that the wild type strain has fully deactivated the repressor complex, but also indicates that neither Rgt1p nor Mth1p can function without the other. Both proteins are equally important in maintaining the repressor complex and the complete loss of either protein results in derepression similar to wild type. The effect of combinations of null mutations of these genes and in combination with of loss of *STD1* on the adaptation time of *snf3* null strains was also evaluated (Figure 3B). Again, the adaptation times were equivalent suggesting that simultaneous mutation of *mth1* and *rgt1* or of *mth1* and *std1* do not display additive effects.

**Figure 4:**
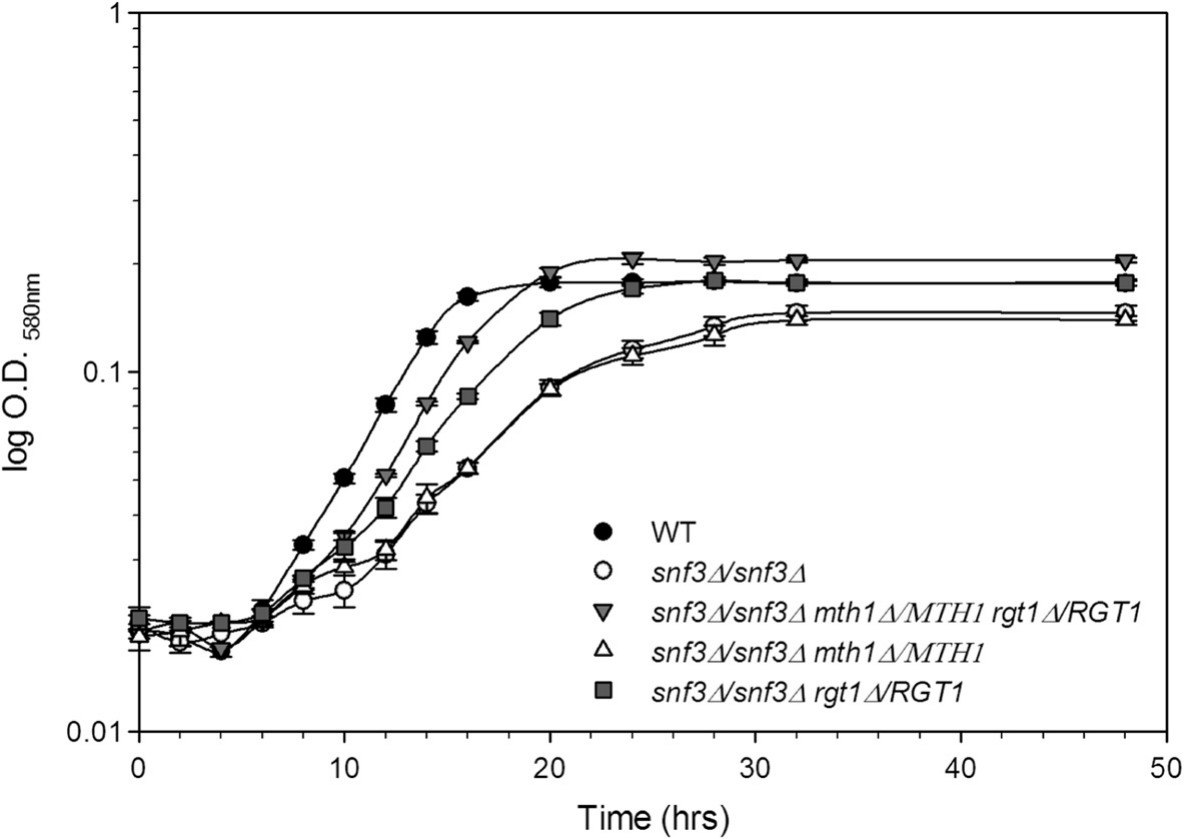
**Low glucose growth curve of diploid yeast strains with single and combined heterozygous alleles of *MTH1 *and *RGT1 *in a homozygous *snf3 *null background demonstrate varying adaptation time differences compared to wild type and homozygous *snf3 *null.** WT (Wild Type) (YPH501), *snf3/snf3* (UCD2882), *snf3/snf3 mth1/MTH1 rgt1/RGT1* (UCD2881), *snf3/snf3 mth1/MTH1* (UCD2879), and *snf3/snf3 rgt1/RGT1* (UCD2880).

The heterozygous diploid strains were also examined during growth on low glucose in liquid media. Both the single *snf3/snf3 rgt1/RGT1* heterozygote and the *snf3/snf3 rgt1/RGT1 mth1/MTH1* double heterozygote decrease the adaptation time as compared to the *snf3/snf3* strain (Figure 4). The single *snf3/snf3 mth1/MTH1* heterozygote displayed a growth pattern identical to *snf3/snf3*, while the *snf3/snf3 rgt1/RGT1* heterozygote had an intermediate effect with the *snf3/snf3 rgt1/RGT1 mth1/MTH1* double heterozygote showing the greatest decrease in adaptation time. The double heterozygote lags behind wild type, but is considerably faster than the *snf3/snf3* null strain or either of the single heterozygotes. This analysis confirms the model of an impact of gene dosage on expression. Reduction of gene dosage would be expected to lead to an intermediate phenotype between complete loss of the complex and its presence at wild type levels, exactly as observed in the growth analyses.

### *The combined haploinsufficiency phenotype is a result of* HXT *gene expression*

Because Mth1p and Rgt1p are known to regulate the expression of many of the *HXT* genes, β-galactosidase reporter assays were used to examine the expression of the *HXT* genes most likely to be relevant to adapting to low glucose conditions. Reporter plasmids with the regions spanning at least −950 up to the start codon of *HXT2, HXT3* and *HXT4* were transformed into diploid strains with different combinations of mutations of components of the pathway. These strains were grown under fully repressing conditions on galactose, shifted to low glucose plus Antimycin A and then harvested and assayed for β-galactosidase activity.

First, all three reporter plasmids were tested in the wild type (YPH501), *snf3/snf3* and *snf3/snf3 rgt1/RGT1 mth1/MTH1* diploids (Table 1) to examine differences in the capacity to regulate these promoters in these strains. All three promoters are strongly repressed in the *snf3/snf3* null diploid and induced to different degrees in the wild type strains. Analysis of reporter gene activity in the *snf3/snf3 rgt1/RGT1 mth1/MTH1* strain demonstrated slight but measurable increases in expression from the *HXT3* and *HXT4* promoters, and a more dramatic 10-fold increase in expression from the *HXT2* promoter (Table 2). The difference in the activity of β-galactosidase reporter assays indicated a clear effect on the transcriptional activity of the high affinity *HXT* gene, *HXT2,* in the presence of heterozygosity at the *MTH1* and *RGT1* loci. In addition, *RGT1* also demonstrated a measurable amount of single haploinsufficiency in growth curves (Figure 4) and reporter assays (Table 2), but the more complete suppression as a result of the combined haploinsufficiency demonstrates that this gene dosage effect not only pertains to the DNA binding repressor, but also to the corepressor protein Mth1p. These observations are consistent with previous work on Rgt1p-mediated repression [26].

**Table 1:** Yeast strains used in this study

**Strain**	**Genotype**	**Source**
YPH500	*MATα*	P. Hieter
YPH501	*MATa/MATα*	“
UCD2875	*MATa snf3Δ4::HIS3 mth1Δ::HphMX*	This Work
UCD2876	*MATa snf3Δ4::HIS3 rgt1Δ::KanMX*	“
UCD2877	*MATa snf3Δ4::HIS3 std1Δ::KanMX*	“
UCD2878	*MATα snf3Δ::TRP1 std1Δ::KanMX*	“
UCD2879	*MATa/MATα snf3Δ4::HIS3/snf3Δ::TRP1 mth1Δ::HphMX/MTH1*	“
UCD2880	*MATa/MATα snf3Δ4::HIS3/snf3Δ::TRP1 rgt1Δ::KanMX/RGT1*	“
UCD2881	*MATa/MATα snf3Δ4::HIS3/snf3Δ::TRP1 mth1Δ::HphMX/MTH1 rgt1Δ::KanMX/RGT1*	“
UCD2882	*MATa/MATα snf3Δ4::HIS3/snf3Δ::TRP1*	“
UCD2883	*MATa/MATα snf3Δ4::HIS3/snf3Δ::TRP1 mth1Δ::HphMX/mth1Δ::HphMX*	“
UCD2884	*MATa/MATα snf3Δ4::HIS3/snf3Δ::TRP1 rgt1Δ::KanMX/rgt1Δ::KanMX*	“
UCD2885	*MATa/MATα mth1Δ::HphMX/mth1Δ::HphMX*	“
UCD2886	*MATa/MATα snf3Δ4::HIS3/snf3Δ::TRP1 mth1Δ::HphMX/mth1Δ::HphMX rgt1Δ::KanMX/rgt1Δ::KanMX*	“
UCD2887	*MATa/MATα snf3Δ4::HIS3/snf3Δ::TRP1 std1Δ::KanMX/std1Δ::KanMX*	“
UCD2888	*MATa/MATα snf3Δ4::HIS3/snf3Δ::TRP1 mth1Δ::HphMX/mth1Δ::HphMX std1Δ::KanMX/std1Δ::KanMX*	“
UCD2889	*MATα snf3Δ::TRP1 hxt2∆ ::KanMX*	“
UCD2890	*MATa snf3Δ::TRP1 hxt2∆ ::KanMX*	“
UCD2891	*MATa snf3Δ::TRP1 hxt2∆ ::KanMX rgt1:HphMX*	“
UCD2892	*MATα snf3Δ::TRP1 hxt2∆ ::KanMX mth1::HphMX*	“
UCD2893	*MATα snf3Δ::TRP1/ snf3∆ ::TRP1 hxt2∆ ::KanMX/ hxt2∆ ::KanMX rgt1:HphMX/RGT1 mth1::HphMX/MTH1*	“

*All strains are *ura3-52, lys2-801, ade2-100, trp1-63 his3Δ leu2Δ.*

**Table 2:** **β-galactosidase activity of *HXT 2, 3 *and *4 *promoter fusions to LacZ**

**Relevant Genotype**	LacZ Fusion Reporter Assay^*^		
	**β-Galactosidase Activity (nmole/min/mg protein)**		
	***HXT2***	***HXT3***	***HXT4***
Wild Type	323.8±7.4a	41.5±3.7b	31.4±1.5c
*snf3/snf3*	2.8±0.07e	1.3±0.07e	1.0±0.37e
*snf3/snf3 rgt1/RGT1 mth1/MTH1*	26.6±1.8d	2.8±0.2e	1.6±0.04e

^*^Strains were grown to mid log phase in SC-URA + 2% galactose and then transferred to SC-URA with 0.05% glucose plus Antimycin A and harvested and assayed 3 hours after the shift. Values represent the average +/− standard deviation of at least three separate transformants cultured separately with the same reporter construct. Different letters denote significant differences at p <0.05.

Because the *HXT2* promoter was the most sensitive reporter to changes in regulatory machinery and promoter copy numbers, it was used to compare the strains that demonstrated some amount of single haploinsufficiency as well as the homozygous nulls of *mth1* and *rgt1* (Table 3). Strains that were grown under repressing conditions on galactose and then shifted back to galactose or to low glucose shows that the wild type strain exhibits some expression of *HXT2* under repressing conditions that is dependent on functional Snf3 along with a strong induction on low glucose (Table 3). The *snf3/snf3 mth1/MTH1 rgt1/RGT1* strain shows leaky expression irrespective of carbon source. The *snf3/snf3 rgt1/RGT1* strain also shows leaky expression irrespective of carbon source, but less than that of *snf3/snf3 mth1/MTH1 rgt1/RGT1,* which is consistent the spot plate assays and growth curves described above. The *snf3Δ/snf3Δ* and *snf3Δ/snf3Δ std1Δ/std1Δ* strains did not display induction of the *HXT2* promoter. The homozygous null diploid strains, *snf3Δ/snf3Δ rgt1Δ/rgt1Δ*, *snf3Δ/snf3Δ mth1Δ/mth1Δ* and *snf3Δ/snf3Δ rgt1Δ/rgt1Δ mth1Δ/mth1Δ* strains showed levels of expression of the *HXT2* promoter much higher than that of wild type likely due to higher basal levels of promoter activity due to reduction of corepressor binding. The low level of expression of the *HXT* genes observed in the heterozygous null strains appears to be responsible for the enhanced growth on low glucose concentrations observed in both the spot plate assays and the low glucose growth curves.

**Table 3:** **β-galactosidase activity expressed from the *HXT2 *promoter fusions of strains following shift to low glucose or galactose**

**Relevant Genotype**	* LacZ *Fusion Reporter Assay^*^	
	** β-Galactosidase Activity (nmole/min/mg protein)**	
	**Glucose Shift**	**Galactose Shift**
Wild Type	196.7±4.6b	21.5±2.7c,a
*snf3/snf3*	1.2±0.3c,c	1.6±0.3c,d
*snf3/snf3 mth1/MTH1 rgt1/RGT1*	9.2±1.0c,a	8.8±0.3c,b
*snf3/snf3 rgt1/RGT1*	4.2±0.7c,b	3.4±0.23c,c
*snf3/snf3 mth1/MTH1*	1.2±0.1c,c	1.1±0.41c,d
*snf3/snf3 std1/std1*	1.8±1.2c,c	1.4±0.67c,d
*snf3/snf3 mth1/mth1*	678±158.5a	589.8±49.6a
*snf3/snf3 rgt1/rgt1*	309.8±25.9b	321.5±12.98b
*snf3/snf3 mth1/mth1 rgt1/rgt1*	563.6±167.7a	574.9±113.5a

^*^Strains were grown to mid log phase in SC-URA + 2% galactose and then transferred to either SC-URA with 2% galactose or to SC-URA with 0.05% glucose plus Antimycin A and harvested and assayed 3 hours after the shift. Values and standard deviation and represent the average of at least three transformants cultured separately with the same reporter construct. Different letters denote statistical significance at p < 0.05. The first set of letters represents differences among values of the entire data set. The second set of letters denotes statistical significance among activity values at or below 20 units.

### *Specific derepression of* HXT2 *is responsible for growth of the heterozygotes*

**Figure 5:**
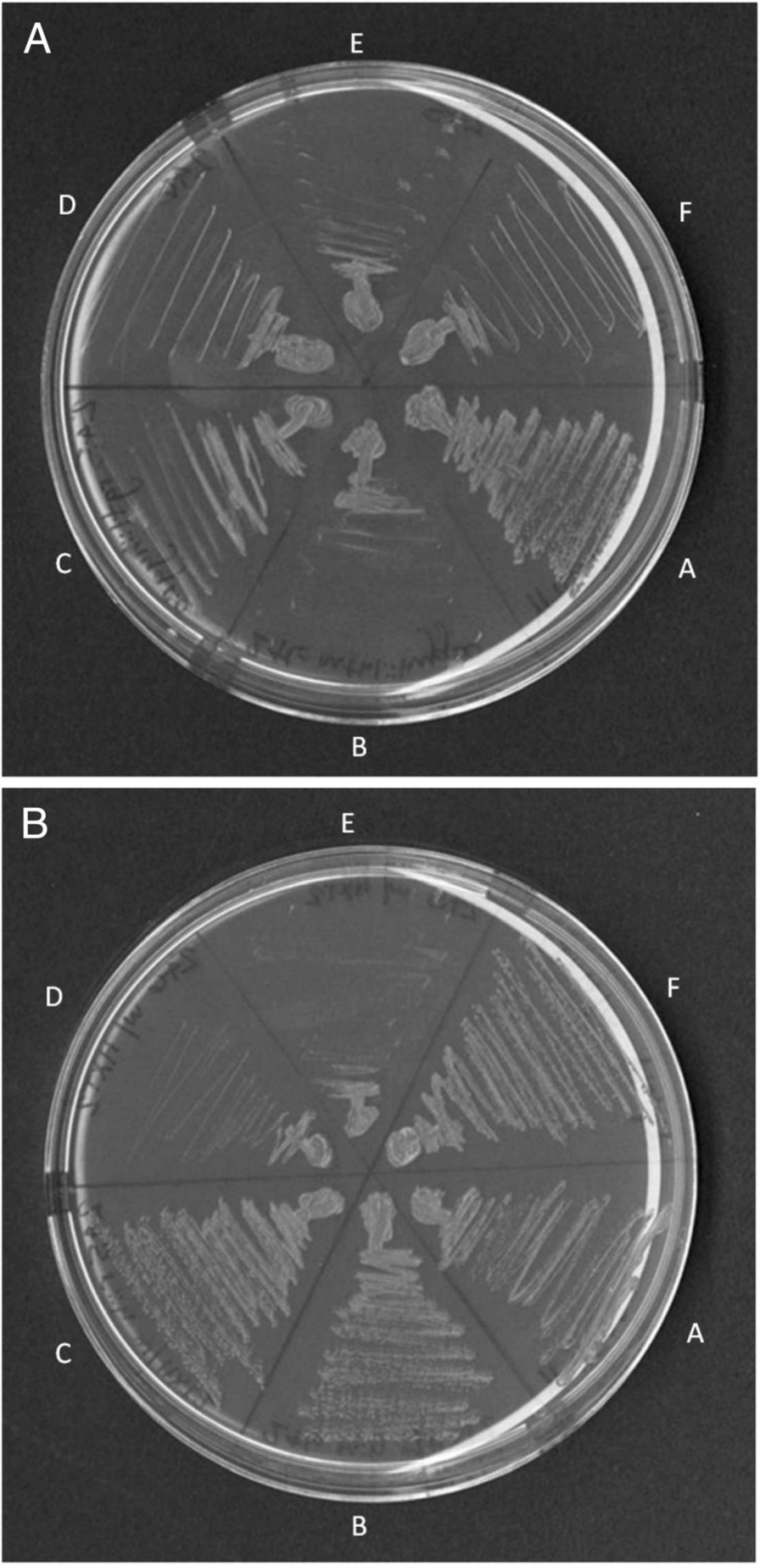
**The *HXT2 *gene is required for the expression of growth in strains heterozygous for *mth1 *and *rgt1.*** Panel **A**: mutant strains. Panel **B**: mutant strains transformed with the *HXT2* gene on a CEN plasmid. **A**: *SNF3 HXT2 MTH1 RGT1* (YPH500); **B**: *snf3∆ hxt2∆ mth1∆ * (UCD2892); **C**: *snf3∆ hxt2∆ rgt1∆ * (UCD2891); **D**: *snf3∆ hxt2∆ * MATα (UCD2889); **E**: *snf3∆ hxt2∆ MATa* (UCD2890); **F**: *snf3∆ /snf3∆ hxt2∆ /hxt2∆ / mth1∆ /MTH1 rgt1∆ /RGT1* (UCD2893).

The analysis of the impact of loss of repressor gene dosage on *HXT* gene expression implicated increased expression of the *HXT2* gene as the mechanism by which glucose transport is restored to the cells. However this analysis was conducted using reporter gene constructs. If the expression of haploinsufficiency is mediated by the *HXT2* gene then suppression of the *snf3* phenotype would be expected to be abolished in a strain carrying a deletion of *HXT2*. To test this possibility, a null mutation of the *HXT2* gene was constructed in the heterozygous diploid (*snf3Δ/snf3Δ RGT1/rgt1Δ MTH1/mth1Δ hxt2Δ/hxt2Δ*). Null of *HXT2* were also constructed in a series of haploid mutations to assess the impact of loss of this transporter on suppression of the *snf3* growth defect in a variety of backgrounds. The *hxt2* null strains were transformed with the *HXT2* gene and used as a control. Loss of the *HXT2* gene did not impact growth of the wild type strain on low glucose, YPH500 (Figure 5, Sector A). Growth of strains carrying the *snf3*∆ mutation was unaffected by the presence of a wild type copy of *HXT2* (Sectors E and F). The growth of both haploid strains, *snf3∆ hxt2∆ mth1∆ * (Sector B) and *snf3∆ hxt2∆ rgt1∆ * (Sector C) was improved by transformation with the *HXT2* gene (Panel B). Growth of the heterozygous diploid, *snf3∆ / snf3∆ hxt2∆ */ *hxt2∆ mth1∆ /MTH1 rgt1∆ /RGT1* (Sector F) was greatly reduced by deletion of the *HXT2* gene and became similar to the *s∆ nf3* mutation (compare sector E to sector F). Thus the expression of combined haploinsufficiency requires the presence of a wild type copy of the *HXT2* gene. The fact that growth of strains carrying the *snf3* suppressors *mth1∆ * and *rgt1∆ * in a haploid state also requires the presence of the *HXT2* gene suggests that expression of this transporter is important for growth under these low glucose conditions.

Analysis of the distribution of Rgt1 binding sites in *HXT* gene promoter regions revealed a direct connection between the number of binding sites and the level of repression. Addition of binding sites to a reporter gene construct increased repression 10 to 50 fold [26]. These authors speculated that Rgt1-mediated repression of genes containing less than five Rgt1-binding sites likely required the presence of another repressor. The *HXT2* promoter was shown to carry only three Rgt1 binding sites while the promoters of the *HXT1* and *HXT3* genes carried 13 and 15 putative binding sites for Rgt1p-mediated repression respectively [26]. Our findings suggest that the combination of both Rgt1 and Mth1 as co-repressors is required for full *HXT2* repression. When considered in combination with the previous reports that 2 micron plasmids containing only the promoters of genes regulated by this complex can suppress the *snf3* growth defect [27], the clear conclusion is that this pathway has evolved a fine balance between the number of promoter binding domains and repressor complex availability. This balance allows for effective gene repression when glucose is not present yet efficient and prompt derepression when glucose is available.

These observations also suggest that repressor binding site number may represent yet another mechanism to differentially regulate the different hexose transporters. The Hxt2 transporter has been shown to exist in forms with differing affinity for substrate [28] although under some conditions it appears to be a simple high affinity transporter [29]. Hxt2p has also been shown to be expressed early in wine fermentation when sugar substrates are in high concentration, but then is quickly degraded [30-32]. These observations suggest that the *HXT2* protein may be an important transporter for the transition between substrate concentrations and that the affinity of the transporter may be impacted by other factors within the cell. It is also possible that under very high substrate conditions the Hxt2 transporter plays an important regulatory role and that putative regulatory role rather than level of transporter protein expression is the key factor enabling growth of the heterozygous strain. A rapid induction of *HXT2* may therefore confer a distinct growth advantage to the cells during adaptation to differing substrate concentrations. Differences in numbers of repressor binding sites could play a role in allowing expression of some transporters to occur under otherwise limiting conditions.

## Conclusions

This information adds unique insight to the model that has formed over the past decade since the roles of the corepressor proteins were first elucidated and this intriguing mechanism of transcriptional regulation began to be understood [11,16,17]. One of the most striking features of this pathway is the apparent large expense of cellular energy required to utilize a signaling system based on the complete degradation of the corepressor proteins Mth1p and Std1p. The observation of combined haploinsufficiency indicates that one mechanism by which this system conserves energy is that just enough corepressor protein is available to maintain repression of the target genes of the pathway. As a result, the system is extremely sensitive and responsive to subtle changes in corepressor numbers. Keeping the level of corepressor protein to the minimum amount necessary to maintain repression allows for quick induction of gene expression when the preferred carbohydrate returns.

## Methods

### Yeast strains, genetic techniques, and growth media

The strains of *Saccharomyces cerevisiae* used in this study are listed in Table 1 above. The MATa deletion set of the *Saccharomyces* Genome Deletion Project was purchased from Open Biosystems and used to make all complete null alleles that contain the *KanMX* resistance marker that provides resistance to G418. Genomic DNA was purified by using the MasterPure™ Yeast DNA purification kit (Epicentre). Genomic DNA of the respective null strain from the deletion set was used as the template for PCR mediated gene disruption by amplifying approximately 200 base pairs of homology flanking each side of the deleted ORF. For all deletions that contain the *HphMX* resistance marker specifying resistance to Hygromycin B, the pYC140 plasmid [33] was used as a template for PCR with primers with at least 40 base pairs of flanking homology just outside of the open reading frame. The linear PCR products were PCR purified (Qiagen) and transformed by the method of Gietz and Woods and selected for with the appropriate antibiotic [34]. All deletions were made in haploid strains and confirmed by colony PCR as described in [35]. Diploids were made by mating the appropriate haploid strains. Standard genetic techniques were used for mating and sporulation.

Yeast strains were grown on Yeast Extract Peptone Media (YP) consisting of 1% yeast extract, 2% Bacto peptone and either 2% glucose, 2% galactose, or 0.05% glucose. Synthetic complete (SC) or synthetic dropout (SD) media were made according to established protocols [35] with the appropriate amino acid or nitrogen base omitted. Bacto agar at 2% w/v was used for solid media. Antimycin A was added at 1 μg/mL to low glucose (0.05%) media. When needed for selection, G418 was used at 200 μg/ml or Hygromycin B used at 300 μg/mL. Growth on low glucose was assessed by streaking strains to YP 0.05% glucose plus Antimycin A or SD with the necessary dropout to maintain selection for the plasmid plus 0.05% glucose and Antimycin A. Growth was assessed after 3 days of incubation at 30°C.

### Construction of overexpression vector for STD1

The complete open reading frame of *STD1* was cloned into the p416TEF vector which contains the *URA3* selectable marker [36] by using PCR primers that introduced 5’ EcoRI and 3’XhoI flanking sites. The respective sites were then used to clone into the vector and the open reading frame was sequenced from the vector to ensure the fidelity of the amplification and cloning. The resulting vector, pKD-TEF-STD1 contains the complete open reading frame of *STD1* preceded by the strong constitutive TEF promoter and flanked by the *CYC1* terminator sequence.

### Fusion constructs

The LacZ reporter fusions to the promoters of *HXT2*, *HXT3*, and *HXT4* were made in the CEN/ARS vector pRS416 which contains the *URA3* selectable marker [37]. The promoter regions spanning at least −950 up to the start codon of *HXT2*, *HXT3* and *HXT4* were PCR amplified with 5’ SacI and 3’NotI overhangs and cloned into pRS416 to create pKD-HXT2, pKD-HXT3 and pKD-HXT4. The resulting plasmids were then sequenced to ensure the fidelity of the cloning. The *E. coli* LacZ gene from the one hybrid reporter vector described in [38] was amplified by PCR with primers that introduced 5’ NotI and 3’ XmaI overhangs and cloned into the respective sites of pKD-HXT2, pKD-HXT3 and pKD-HXT4 to make pKD-HXT2 LacZ, pKD-HXT3 LacZ and pKD-HXT4LacZ. The resulting plasmids contain at least 950 base pairs of the promoter region up to the start codon and are in frame with the *E. coli LacZ* gene. All plasmids were transformed into the respective yeast strains as described in [34].

### *Reintroduction of* HXT2 *on a plasmid*

A plasmid carrying a copy of *HXT2* from YPH500 was constructed in the CEN/ARS vector pRS316, which contains the *URA3* yeast selectable marker [37]. The *HXT2* gene, along with 562bp of the upstream region, was PCR amplified using HiFi Taq (Invitrogen #11304-011) with primers containing 5’ XhoI (5’-CTCGAGTTTCCGTGAAATAGATTC-3’) and 3’ XbaI (5’-TCTAGATATTAGTAGCCATTAGCC-3’) overhangs. The resulting PCR product was cloned into the MCS of pRS316. The plasmid, now carrying a functional copy of *HXT2* under its native promoter, was transformed into previously *hxt2* null strains [34].

### Low glucose growth curves

Strains were streaked out from glycerol stocks to YPD media and a separate single colony was used to inoculate three replicate starter cultures in Synthetic Complete (SC) 2% glucose media. Starter cultures were grown for 48 hours and used to inoculate 200 mL of SC 0.05% glucose plus Antimycin A to an OD580 of 0.02. Cultures were grown in 500 mL non baffled Erlenmeyer flasks at 30°C with shaking on an orbital shaker at 200 RPM and the OD monitored at 580 nm. All strains were examined in triplicate and each figure represents one experiment where all strains shown were grown at the same time. Error bars indicate the standard deviation of the average of the three triplicates.

### Spot plate assays

Overnight cultures of strains were grown in YP 2% glucose, washed once with sterile water and then re-suspended in sterile water to an OD580 of 1.0. The strains were then subjected to a 10 fold serial dilution and 10 μL of each was spotted on YP or SC plus 0.05% glucose and Antimycin A. Growth was assessed after 3 days of incubation at 30°C.

### LacZ reporter assays

Strains with reporter plasmids were grown to mid log phase in SD minus uracil with 2% galactose, harvested by centrifugation and then resuspended in either SD minus uracil with 0.05% and Antimycin A or SD minus uracil with 2% galactose and allowed to grow for 3 hours in the respective media. Approximately 15 OD units were harvested by centrifugation and resuspended in assay buffer and assayed as described in [39]. β-galactosidase units were measured as nmoles of 2-nitrophenyl-β-galactopyranoside released per minute per mg of total protein as determined by Bradford Assay (Biorad). All assays were performed in triplicate and comparisons made between strains that were cultured and assayed at the same time. T-test (LSD) statistical analyses of the *LacZ* data were performed using SAS (SAS Institute Inc., Cary, NC).

## Competing interests

There are no financial or non-financial competing interests for any of the authors.

## Authors’ contributions

KLD and LFB planned all experiments. KLD did the work with the exception of the data provided in Figure 5 which was conducted by EEM. VR assisted in creation of some of the figures and is responsible for the statistical analysis. KLB wrote the original draft. LFB wrote subsequent and final drafts with editing by all authors. All authors read and approved of the final version.

## Authors’ information

KLD current address: Amyris Biotechnologies, 5885 Hollis Street, Suite 100, Emeryville, CA
